# Functional analysis of new human Bardet-Biedl syndrome loci specific variants in the zebrafish model

**DOI:** 10.1038/s41598-019-49217-7

**Published:** 2019-09-10

**Authors:** Sheila Castro-Sánchez, Paula Suarez-Bregua, Rossina Novas, María Álvarez-Satta, Jose L. Badano, Josep Rotllant, Diana Valverde

**Affiliations:** 1 Grupo de Biomarcadores Moleculares, Departamento de Bioquímica, Genética e Inmunología, Facultad de Biología, Universidad de Vigo, Lagoas-Marcosende s/n, 36310 Vigo, Spain; 2 Grupo de Investigación en Enfermedades Raras y Medicina Pediátrica, Instituto de Investigación Sanitaria Galicia Sur (IISGS), Vigo, Spain; 3 Centro de Investigaciones Biomédicas (CINBIO), Centro Singular de Investigación de Galicia 2016–2019, Universidad de Vigo, Vigo, Spain; 4 Department of Biotechnology and Aquaculture, Institute of Marine Research, Spanish National Research Council (IIM-CSIC), Vigo, Spain; 5 Human Molecular Genetics Laboratory, Institut Pasteur de Montevideo, Mataojo 2020, Montevideo, CP11400 Uruguay

**Keywords:** Disease genetics, Mutation

## Abstract

The multiple genetic approaches available for molecular diagnosis of human diseases have made possible to identify an increasing number of pathogenic genetic changes, particularly with the advent of next generation sequencing (NGS) technologies. However, the main challenge lies in the interpretation of their functional impact, which has resulted in the widespread use of animal models. We describe here the functional modelling of seven *BBS* loci variants, most of them novel, in zebrafish embryos to validate their *in silico* prediction of pathogenicity. We show that target knockdown (KD) of known *BBS (BBS1*, *BB5 or BBS6)* loci leads to developmental defects commonly associated with ciliopathies, as previously described. These KD pleiotropic phenotypes were rescued by co-injecting human wild type (WT) loci sequence but not with the equivalent mutated mRNAs, providing evidence of the pathogenic effect of these *BBS* changes. Furthermore, direct assessment of cilia located in Kupffer’s vesicle (KV) showed a reduction of ciliary length associated with all the studied variants, thus confirming a deleterious effect. Taken together, our results seem to prove the pathogenicity of the already classified and unclassified new *BBS* variants, as well as highlight the usefulness of zebrafish as an animal model for *in vivo* assays in human ciliopathies.

## Introduction

Cilia are microtubule-based structures projected from the surface of almost all differentiated cells. These highly conserved organelles can be classified as motile and non-motile (also known as primary cilia). Motile cilia are involved in cell motility, mainly moving extracellular fluids^1,2^, while primary cilia function as sensory cellular antennae for a wide range of signalling pathways such as Sonic hedgehog (Shh), Wnt, planar cell polarity (PCP) and/or Notch, which are essential during embryonic development and also in adult physiology^3,4^. Given the great variety of functions performed by these organelles as well as their presence in nearly all mammalian cells, defects in ciliary axonemal structure and/or cilia function can result in a widespread range of phenotypes leading to human genetic disorders known as ciliopathies^5–7^.

Bardet-Biedl syndrome (BBS; MIM #209900) is an autosomal recessive ciliopathy associated with defects in primary cilia^8^. A wide spectrum of clinical symptoms are observed in BBS patients, among which retinal dystrophy, obesity, limb abnormalities, cognitive impairment, renal and urogenital anomalies have been classified as primary features. A number of secondary features, such as diabetes mellitus type 2, developmental and psychomotor delay, cardiovascular anomalies and craniofacial defects, are frequently observed. Pathogenic variants in 21 genes (*BBS1-21*) have been related to this syndrome, which account for nearly 80% of BBS patients^9,10^.

These genes encode proteins mainly localized to basal bodies and centrosomes, but can also be found in the ciliary axoneme and the transition zone (reviewed in Novas *et al*.^11^). BBS proteins are necessary to maintain primary cilia structure and function^12^, some of them through multiprotein complexes. Such is the case of the BBSome, composed by eight BBS proteins (BBS1, BBS2, BBS4, BBS5, BBS7-BBS9 and BBIP10/BBS18), which participates in the vesicle trafficking of membrane proteins to and inside the primary cilium to promote ciliogenesis^13,14^. The BBSome assembly is mediated by the BBS-chaperonin complex, formed by BBS6, BBS10 and BBS12 proteins^15,16^, together with CCT/TRiC chaperonins. Therefore, altered or non-functional BBS proteins often entail consequences in cilia formation and/or maintenance.

The high genetic and phenotypic heterogeneity typically associated with this syndrome, together with the overlapping phenotypes among ciliopathies which hinder clinical assessment, have made molecular diagnosis a difficult task in many cases. Because of that, researchers are taking advantage of the great variety of current molecular approaches, attempting to achieve a rapid and accurate diagnosis for these patients. In this sense, it is clear that the NGS technology has revolutionised the field of molecular diagnosis and is increasingly being implemented in biomedical research and clinical practice. However, these methods generate a large number of genomic variations whose interpretation represents a great challenge.

This fact points out the importance of performing *in vivo* assays, which are crucial to gain more knowledge about the mechanisms underlying human ciliopathies and to functionally evaluate genetic variants. Several model organisms have been extensively used to study the complex genetic basis of this group of disorders^17^. Although each model has strengths and limitations, vertebrate models have been shown to be more advantageous, mainly to investigate the abnormal organogenesis associated to human ciliopathies^17,18^.

During the last decade, zebrafish (*Danio rerio*) has emerged as a powerful vertebrate model organism for biomedical research, allowing us to interpret the pathogenicity of genetic alterations identified in ciliopathy patients^1,18^. This is because zebrafish offers numerous advantages compared with other animal models such as low cost, rapid development, embryonic transparency and easy manipulation, as well as large fecundity rates with clutches of several hundred eggs. In addition, the availability and relatively easy-handling of antisense KD techniques contribute to the understanding of gene function. Particularly, morpholino antisense oligonucleotides (MOs), when properly controlled for off-target effects and toxicity, have been shown to be a powerful tool to study gene function^1,19,20^.

The zebrafish model has several ciliated organs containing both primary and motile cilia^1^. KD of *BBS* genes in zebrafish are reported to cause early developmental phenotypes typically associated with PCP pathway defects^21,22^. These are usually initiated along with KV disruption, a transient ciliated organ that, when affected, leads to defects in left-right asymmetry establishment, the first embryonic process related to cilia function^2^.

Here we report the functional characterization of several new *BBS* variants identified in five unrelated patients clinically diagnosed with BBS. *In vivo* assays were performed in zebrafish by combining antisense MO gene KD approach and human mRNA for rescue experiments to assess developmental defects during gastrulation, particularly in KV.

## Results

### Molecular genetic diagnosis

The use of different genetic tools (genotyping microarray, direct sequencing, homozygosity mapping, and whole exome sequencing –WES-) led us to identify seven candidate variants in three *BBS* genes in this group of patients clinically diagnosed with BBS (shown in Table 1). Three of the *BBS1* (MIM #209900) variations (except p.Met390Arg) were previously reported as novel by our group^23^ and included in this study for functional characterization. The missense change in this gene (p.(Val366Asp)) has been predicted to be pathogenic by four bioinformatics tools (Table 2). The nonsense and deletion variants have been assumed as pathogenic.

**Table 1:** Genetic and phenotypic data of the patients under study.

Patient	Gender	Origin	Gene	Variant	Reported study	RD	OB	PD	UA	CI	RA	Other
GBB22	F	Asian (India)	*BBS1*	p.(Trp23*)/p.(Trp23*)	^23^	+	+	+	NA	NA	NA	−
RP1377	F	Caucasian (Spanish)	*BBS1*	p.(Val3661Asp)/p.(Val366Asp)	^23^	+	+	+	NA	−	−	CC
GBB27	M	Caucasian (Spanish)	*BBS1*	p.Met390Arg/p.(His504Hisfs*48)	^23, 58^	+	+	+	NA	+	−/NA	HL, CA
RP1573^^^	F	Caucasian (Spanish)	*BBS5/BBS6*	p.Asn184Ser/wt p.(Gly411Ala)/p.(Gly411Ala)	Ref.^29^/This study	+	+	+	−	+	+	CAT, H, HT, D, GL
RTP23	M	Caucasian (Spanish)	*BBS5*	p.(Arg138Cys)/wt wt/p.(Phe180Phefs*6)	This study (both)	+	+	+	+	+	−	CA

p.(Arg138Cys) variant was classified as novel in this study since it was not found un public databases at the moment of identification. Abbreviations: F, female; M, male; RD, retinal dystrophy; OB, obesity; PD, polydactyly; UA, urogenital anomalies; CI, cognitive impairment; RA, renal abnormalities; CC, congenital cardiomyopathy; HL, hearing loss; CA, craniofacial anomalies; CAT, cataracts; H, hypothyroidism; HT, hypertension; D, dyslipidemia; GL, Glaucoma. ^(^)^Consanguineous family. Reference gene sequences: NG_009093.1 for *BBS1*, NG_011567.1 for *BBS5*, and NG_009109.1 for *MKKS/BBS6*.

**Table 2:** Pathogenicity prediction at protein level of all variants by several *in silico* tools.

GENE	VARIANT	PolyPhen-2	SIFT	Pmut	Mutation Taster	SCORE
*BBS1*	p.(Trp23*)	*Pathological*				—
	p.(Val366Asp)	Possibly damaging	Damaging	Pathological	Disease causing	4/4
	p.(His504Hisfs*48)	*Pathological*				—
*BBS5*	p.(Arg138Cys)	Probably damaging	Damaging	Pathological	Disease causing	4/4
	p.(Phe180Phefs*6)	*Pathological*				—
	p.(Asn184Ser)	Probably damaging	Damaging	Neutral	Disease causing	3/4
*BBS6*	p.(Gly411Ala)	Probably damaging	Damaging	Neutral	Disease causing	3/4

Nonsense and frameshift variants were considered directly as pathological/damaging. Reference sequences used for predictions (*Swiss-Prot/Ensembl*): Q8NFJ9/ENSP00000317469 for *BBS1*, Q8N3I7/ENSP00000295240 for *BBS5* and Q9NPJ1/ENSP00000246062 for *BBS6*.

**Figure 1:**
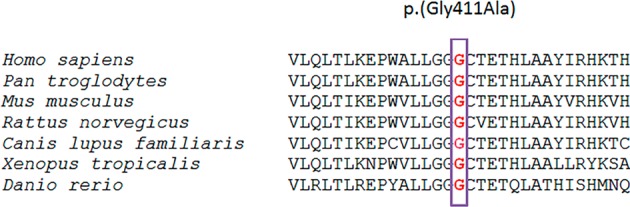
Alignment of a fragment of BBS6/MKKS protein showing complete conservation of residue 411 across species. *Homo sapiens* (human), *Pan troglodytes* (chimpanzee), *Mus musculus* (mouse), *Rattus norvegicus* (rat), *Canis lupus familiaris* (dog), *Xenopus tropicalis* (frog), *Danio rerio* (zebrafish).

**Figure 2:**
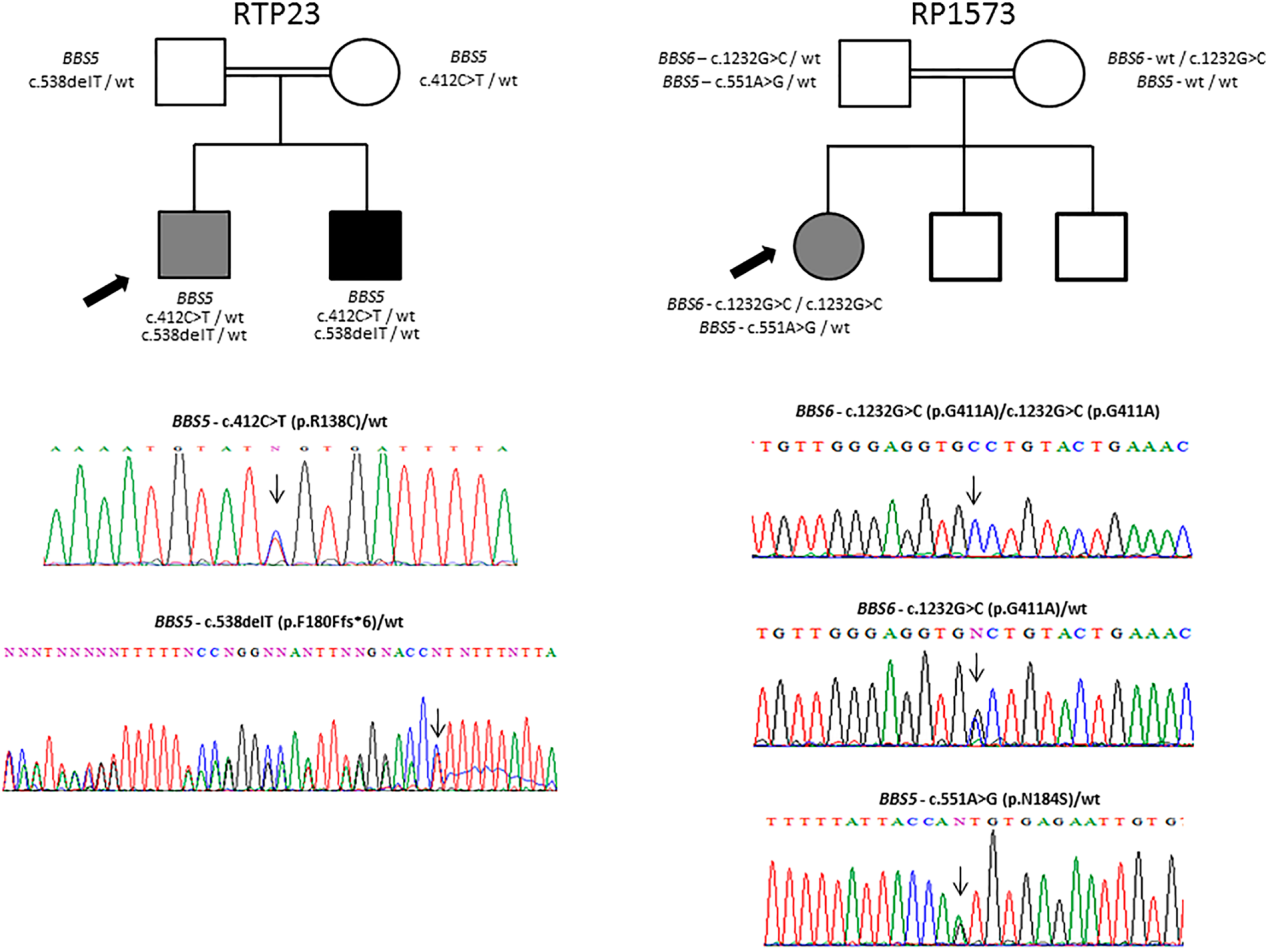
Segregation of the variants identified in *BBS5* and *BBS6* genes.

Homozygosity mapping, performed in the only patient belonging to a consanguineous family (RP1573), combined with direct sequencing revealed a novel homozygous missense change (p.(Gly411Ala)) in *MKKS/BBS6* (MIM #604896) gene (Table 1), which has been predicted to be pathogenic by three out of four bioinformatics tools (Table 2), localizes in a highly conserved region of the encoded protein (Fig. 1) and segregates from both parents (Fig. 2). On the other hand, two variants in heterozygous state (p.(Arg138Cys) and p.(Phe180Phefs*6)) were identified by WES in *BBS5* (MIM #603650) gene in patient RTP23. All pathogenicity tools provided a damaging prediction for the missense change (Table 2). The novel deletion was assumed to be pathogenic. Both have been validated by direct sequencing and segregate within the family (Fig. 2). We also analysed *in silico* their potential effect on splicing, finding that all of them have a positive prediction with at least two out of four tools, either modifying or eliminating a donor or acceptor splice site (Table 3). Novel variants were absent in 100 control alleles of Galician origin, and their frequency was checked in several public databases.

**Table 3:** Impact prediction of *BBS* variants on splice sites, an indicative of possible splicing defects.

VARIANT	*NetGene2*	*NNsplice*	*HSF*	*Rescue ESE*	Score
***BBS1***					
c.68 G > A/p.(W23*)	+	**−**	+	+	3/4
c.1097 T > A/p.(V366D)	+	**−**	**+**	+	3/4
c.1510_1520delCACCTGCAGAA/p.(H504Hfs*48)	+	+	+	+	4/4
***BBS5***					
c.412 C > T/p.(R138C)	+	**−**	+	**−**	2/4
c.538delT/p.(F180Ffs*6)	+	+	+	**−**	3/4
c.551 A > G/p.(N184S)	**−**	**+**	+	**−**	2/4
***BBS6/MKKS***					
c.1232 G > C/p.(G411A)	**−**	**−**	+	**−**	1/4

(+) positive prediction; (**−**) negative prediction. Protein change abbreviated nomenclature (one-letter code for each amino acid, used in Fig. 3).

### Whole-mount *in situ* hybridization reveals early developmental defects in zebrafish

According to the previous evidences of the potential pathogenicity of the identified variants, their functional effect was evaluated *in vivo*. To do this, zebrafish embryos (n = 180–300 embryos/MO) were first injected with specific MOs against each *bbs* gene to assess KD phenotypes at 8–12 somite stage. The specificity and efficacy of the MOs used in this work have already been established in a previous study^24^. Thus, consistent with published data^24^, our results also show that among the MOs-injected animals, 97% showed several gastrulation defects commonly associated with BBS phenotypes, including shortened body axis/length, kinked and wide notochords, and thinner somites (Fig. 4).

Co-injection of each *bbs*-MO with the corresponding WT human *BBS* capped-mRNA efficiently rescued those morphant phenotypes, which confirms the specificity of the MOs targeting. Thus, the defects associated with planar cell polarity (PCP), as indicated by various gene markers, were almost completely rescued by wildtype human mRNA injection.

Then, we carried out the individual rescue experiments by injecting embryos (n = 180–300 embryos/variant) with the *bbs*-MO plus mutant human *BBS* capped-mRNA to assess their ability of *BBS* variants to rescue the MO-associated phenotypes. Expression and translation stability of mutated human BBS proteins (from RNA transcripts) from their DNA constructs used in the individual rescue experiments was assessed in an *in vitro* transcription and translation-coupled system (TNT^®^ Coupled Reticulocyte Lysate Systems; Promega). Figure 3 illustrates the *in vitro* transcription/translation assay using the WT and mutant human cDNA constructs showing that each pCS + -cDNA construct adequately translated and labelled into a biotinylated protein. The kDa size of the each *in vitro* translated protein accurately correlates with the expected ORF (Fig. 3), therefore, indicating the transcriptional stability of the different mutated human DNA constructs generated.

**Figure 3:**
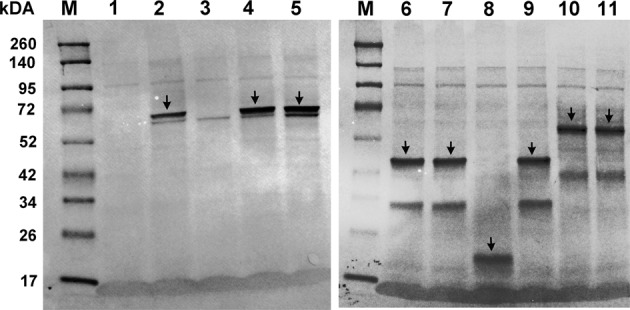
Expression and translation stability of WT and mutated human BBS proteins (from RNA transcripts) from their DNA constructs in an *in vitro* transcription and translation assay (TNT). Protein products were analyzed using western blot analysis. Resulting proteins were characterized by size and correlated to the size and sequence of a given gene or ORF. The kDa size of the each *in vitro* generated protein correlates exactly with the expected ORF (arrow heads), therefore, indicating the transcriptional stability of the different mutated DNA constructs generated. Non-mutated proteins were used as internal controls (Lanes 2, 6 and 10). Due to the low molecular weight of the expected mutated protein (2 kDa), no band were detected on lane 3. Note: Lane M: standard protein marker; Lane 1: negative control; Lane 2: WT BBS1; Lane 3: BBS1:p.(W23*) mutation; Lane 4: BBS1 p.(V366D) mutation; Lane 5: BBS1 p.(H504fs*48) mutation; Lane 6: WT BBS5; Lane 7: BBS5 p.(R138C) mutation; Lane 8: BBS5:p.(F180fs*6) mutation; Lane 9: BBS5(N184S) mutation; Lane 10: WT BBS6; Lane 11: BBS6:p.(G411A) mutation.

**Figure 4:**
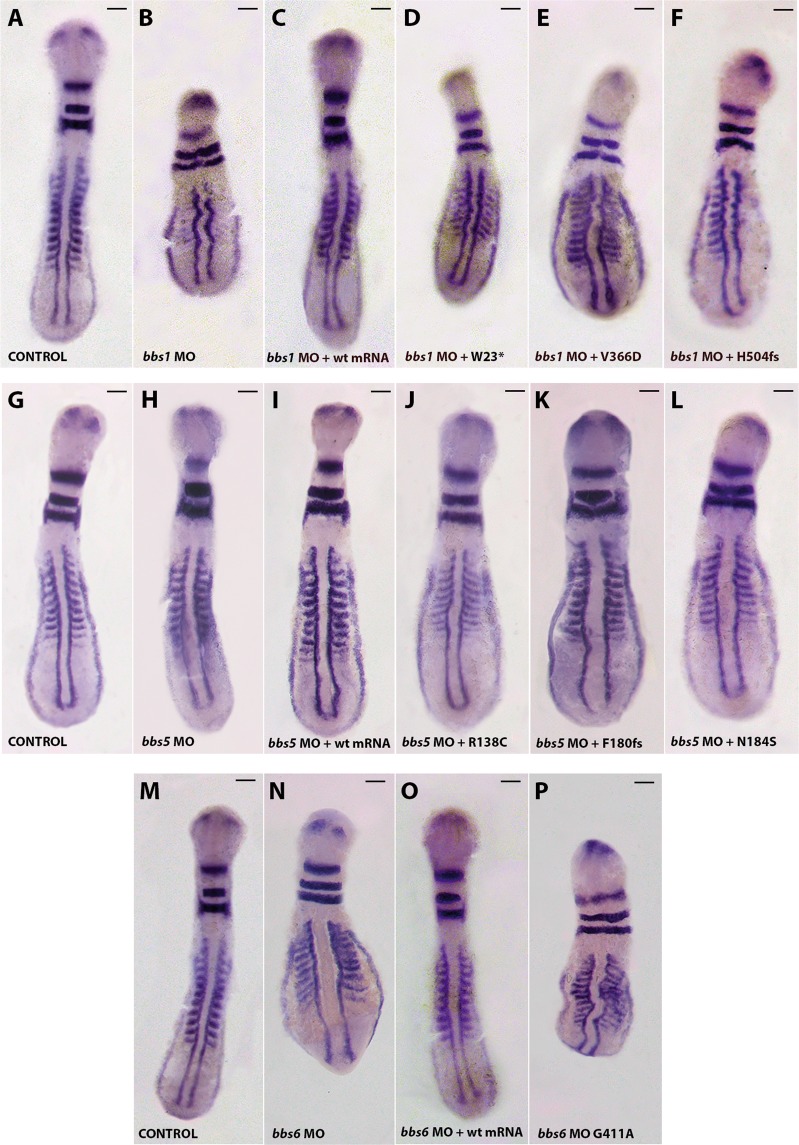
Phenotypes of zebrafish embryos at 8–12 ss, after whole mount *in situ* hybridization. Knockdown of zebrafish *bbs1* (**A**–**F**), *bbs5* (G–L) and *bbs6* (**M**–**P**) genes affects body axis/length, notochord and somite morphology. Morphology of the controls (**A**,**G**,**M**; dorsal view anterior to the top), morpholino (**B**,**H**,**M**; dorsal view anterior to the top), morpholino plus WT human *BBS* capped-mRNA (**C**,**I**,**O**), and morpholino plus sense capped-mRNA of different human BSS variants (**D**–**F**,**J**–**L**,**P**; dorsal view anterior to the top) zebrafish at 12 somites (ss). The embryos were fixed for *in situ* hybridization of *myoD/krox20/pax2*. Injection of WT human BSS1, BSS5 and BSS6 capped-mRNA rescues the phenotype of *bbs1*, *bbs5* and *bbs6* MO-injected embryos, respectively. However, the mutated capped-mRNA of the BBS particular changes of each loci (W23*, V366D, H504fs, R138C, F180fs, N184S, G411A – abbreviated nomenclature) were not able to rescue the respective phenotype of BBS1, BBS5 and BBS6 MO-treated embryos. Scales bars: 100 µm.

To validate the defects observed in live embryos and classify them, we performed *in situ* hybridization with a cocktail of *krox20*, *myoD* and *pax2* riboprobes and examined embryonic defects on whole embryos flat-mounted at 8–12 ss (n = 60–75) (Fig. 4 and Supplementary Fig. S2). Each coinjection of *bbs*-MO along with mutant human capped-mRNA was compared with the MO alone and/or with the respective WT rescue. The percentage of rescued embryos was 95%, 93% and 92% for *bbs*-MO plus BBS1, BBS5 or BB6 WT mRNA coinjections, respectively (Supplementary Fig. S2).

We found that none of the human *BBS* variants were able to entirely recover the zebrafish knockdown *bbs* phenotypes, thereby ruling out the presence of benign variations among the seven *BBS* gene variants studied. Despite of that, some new identified *BBS* variants (e.g., p.(Arg138Cys)) were able to partially rescue the different zebrafish MO-phenotypes features (see Fig. 4). Then, we classified as potentially hypomorphic those changes which rescued some of the different MO phenotype features but showed a more severe phenotype than WT rescue fish, as functional null when similar to MO, and dominant negative when worse than MO. The displayed phenotypes of embryos hybridized *in situ* (shown in Fig. 4) are characterized by several gastrulation defects: shortened body length/axis (especially in *bbs1* and *bbs6* morphants), smaller head (e.g., *BBS1*:p.(Trp23*)), wide and kinked notochord (in nearly all morphants), longer and thinner somites (e.g., *BBS1*:p.(Val366Asp), *BBS5*:p.(Arg138Cys)), partial loss of somite definition (e.g., *BBS1*:p.(His504Hisfs*48), *BBS6*:p.(Gly411Ala)) and reduced eye labelling, suggesting smaller eye size (e.g., *BBS1*:p.(Trp23*) and p.(Val366Asp)). The most efficient mutant rescue, but considerably different from WT rescue, was associated to *BBS5*:p.(Arg138Cys), while *BBS1* and *BBS6* changes seemed to produce the more severe phenotypes. The classification of all variants based on *in situ* results is showed in Table 4.

**Table 4:** Classification of variants based on WISH results.

GENE	VARIANT	Prediction of pathogenicity	Displayed phenotypes
*BBS1*	p.(Trp23*)	Null	Shortened body axis, smaller head, smaller eye size, thinner and longer somites, slightly kinked notochord
	p.(Val366Asp)	Hypomorph	Shortened body axis, smaller eye size, longer somites, slightly kinked notochord
	p.(His504Hisfs*48)	Hypomorph	Shortened body axis, partial loss of somite definition, slightly kinked notochord
*BBS5*	p.(Arg138Cys)	Hypomorph	Longer somites, slightly kinked notochord
	p.(Phe180Phefs*6)	Null	Longer somites, partial loss of somite definition, wide and slightly kinked notochord
	p.(Asn184Ser)	Null	Shortened body axis, smaller head, longer somites, wide notochord
*BBS6/MKKS*	p.(Gly411Ala)	Dominant negative	Shortened body axis, longer and thinner somites, partial loss of somite definition, markedly kinked notochord

### Direct assessment of cilia located in Kupffer’s vesicle (KV) confirms the deleterious effect of new BBS variants

**Figure 5:**
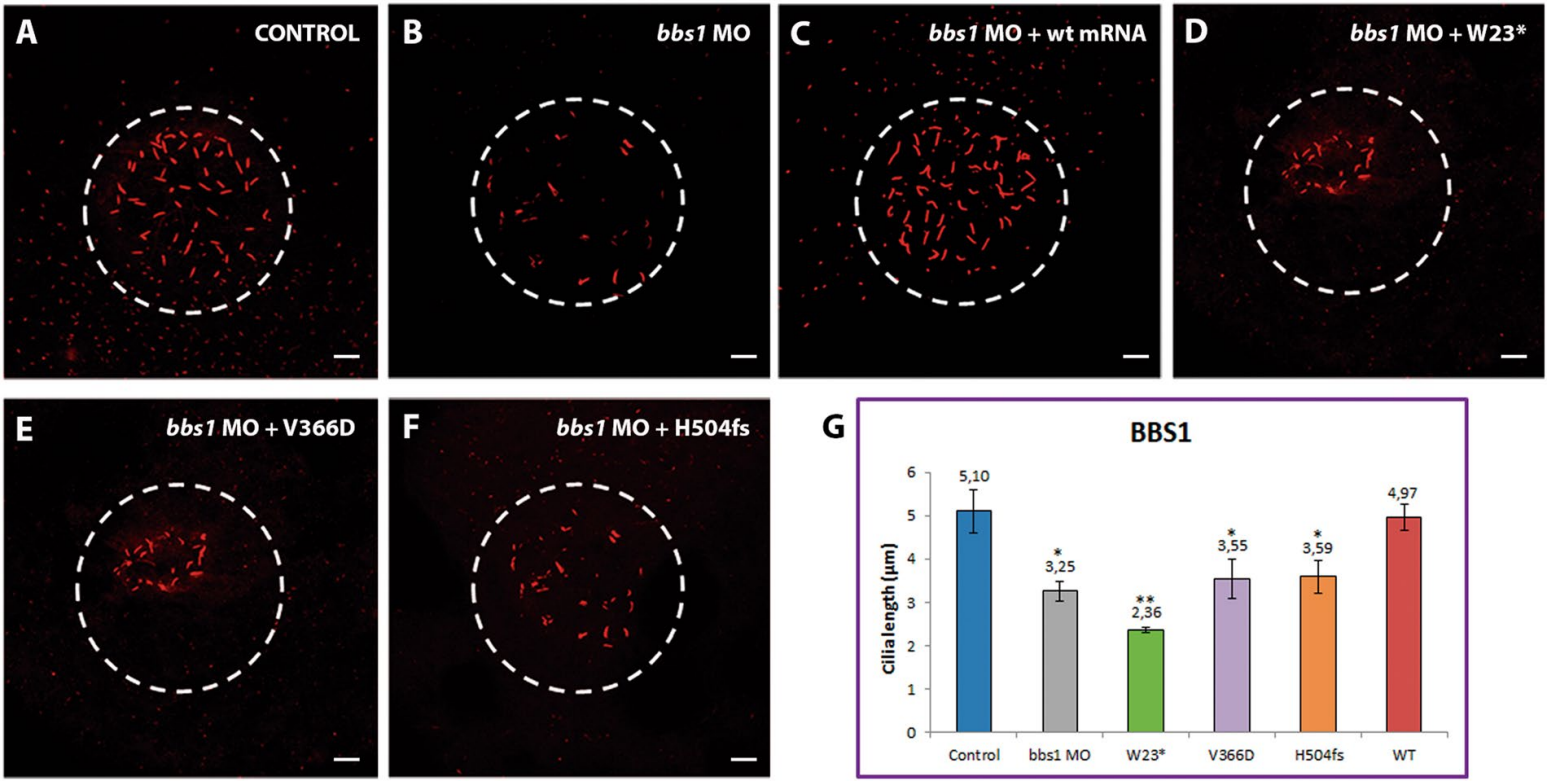
Representative images of KVs and comparison of average cilia length corresponding to *BBS1* conditions. Projections of confocal z-stacks of Alexa fluor 594 and Dapi stained cilia located in Kupffer’s vesicle (KV) from control (**A**) and *bbs* MO-injected embryos (**B**), *bbs* MO-injected embryos plus WT capped-mRNA (**C**) and *bbs* MO-injected embryos plus *BBS1* new changes (W23*, V366D, H504fs) capped-mRNA. (**D**–**F**) Note the reduction of the cilia in *bbs1*-MO- and *BBS1* variants capped-mRNA co-injected embryos. (**G**) Comparison of average cilia length. *p < 0.01, **p < 0.001. Scales bars: 10 µm.

**Figure 6:**
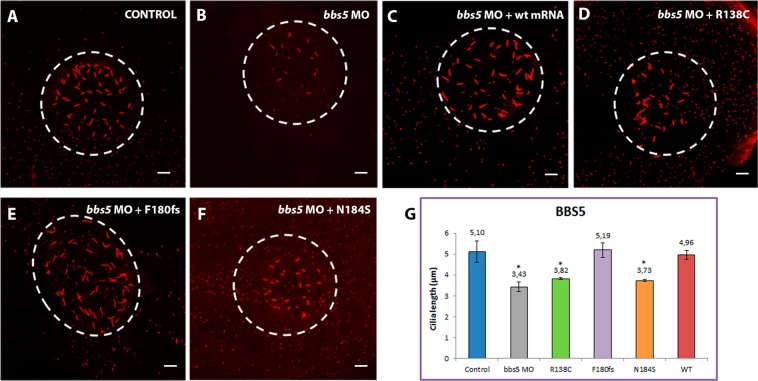
Representative images of KVs and comparison of average cilia length corresponding to *BBS5* conditions. Projections of confocal z-stacks of Alexa fluor 594 and Dapi stained cilia located in Kupffer’s vesicle (KV) from control (**A**) and *bbs5* MO-injected embryos (**B**), *bbs5* MO-injected embryos plus WT capped-mRNA (**C**) and *bbs5* MO-injected embryos plus *BBS5* new changes (R138C, F180fs, N184S) capped-mRNA. (**D**–**F**) Note the reduction of the cilia in *bbs5* MO and *BBS5* variants R138C and N184S capped-mRNA co-injected embryos. (**G**) Comparison of average cilia length. *p < 0.01, **p < 0.001. Scales bars: 10 µm.

**Figure 7:**
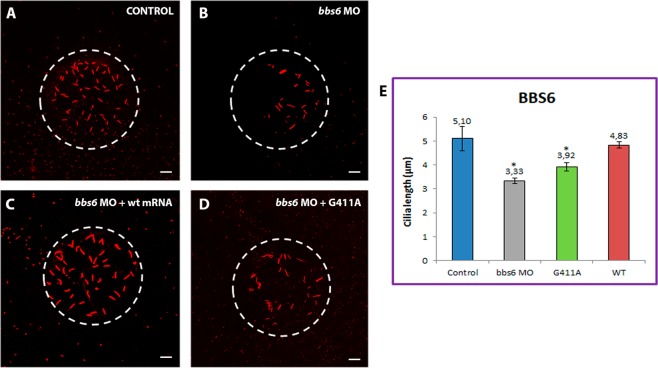
Representative images of KVs and comparison of average cilia length corresponding to *BBS6* conditions. Projections of confocal z-stacks of Alexa fluor 594 and Dapi stained cilia located in Kupffer’s vesicle (KV) from control (**A**) and *bbs6* MO-injected embryos (**B**), *bbs6* MO-injected embryos plus WT capped- mRNA (**C**) and *bbs6* MO-injected embryos plus *BBS6* new changes (G411A) capped-mRNA. (**D**) Note the reduction of the cilia in *bbs6* MO-injected and *BBS6* variant capped-mRNA co-injected embryos. (**E**) Comparison of average cilia length. *p < 0.01, **p < 0.001. Scales bars: 10 µm.

We analysed zebrafish Kupffer’s vesicle (KV) cilia of embryos at 8–10 (±1) somite stage. About 40–45 cilia except in cases of reduced number of cilia, from 5 KVs were measured for each condition to compare with controls. We calculated the average cilia length with associated standard error (standard deviation) and *p*-value (Welch’s *t- test*). As previously reported for other *bbs* genes^25^, KD of *bbs1*, *bbs5* and *bbs6* resulted in a significant reduction in cilia length, from 5.10 + 0.42 µm in controls to 3.25 + 0.22 µm, 3.43 + 0.24 µm and 3.33 + 0.11 µm in *bbs1*, *bbs5* and *bbs6* MO-injected embryos, respectively (*p* < 0.01; Figs 5C, 6C and 7C, respectively). *bbs*-MOs and WT human *BBS* capped mRNA co-injected embryos showed KV cilia length similar to controls (4.97 + 0.31 µm, 4.96 + 0.20 µm and 4.83 + 0.13 µm in *bbs1*, *bbs5* and *bbs6 genes*; *p* > 0.05; Figs 5B, 6B and 7B), confirming the rescue of the morphant phenotype in all cases. Then, we compared *bss* morphants to controls and found that all injected capped mRNA mutant variants resulted in shorter KV cilia (values shown in Figs 5G, 6G and 7E), with the exception of *BBS5*:p.(Phe180Phefs*6), which in contrast fully rescued the morphant phenotype, being not statistically different from controls (Fig. 6G). *BBS1*:p.(Trp23*) change was associated with the most significant shortening of KV cilia, averaging 2.36 + 0.07 µm, even almost 1 µm shorter than *bbs1MO* condition (Fig. 5D,G).

KV cilia are normally organised in a spherical pattern, which can be clearly observed in both controls and WT injected embryos. However, this distribution was disrupted in most morphants, in addition to differences in cilia number. MOs against *bbs1*, *bbs5* and *bbs6* genes led to a significant reduction in cilia number (Supplementary Fig. S1) together with a loss of the spherical pattern (Figs 5C, 6C and 7C, respectively). Overall, *bbs1* morphants showed the most significant KV defects (Fig. 5D–F), with cilia number markedly reduced in all conditions (Supplementary Fig. S1), in addition to the greatest shortening mentioned above. It is noteworthy that *BBS1*:p.(Trp23*) morphant cilia appeared to aggregate into a smaller domain (Fig. 5D). Although no significant differences in cilia length of *BBS5*:p.(Phe180Phefs*6) rescued morphants were found, KV cilia appear to be dispersed, covering a slightly broader area compared to controls (Fig. 6E), as well as a significant reduced number of KV cilia (Supplementary Fig. S1). Interestingly, *BBS6*:p.(Gly411Ala) non-rescued morphant fish also showed a portion of cilia with a curvy shape, not as frequently seen in other morphants (Fig. 7D), also accompanied with a significant lower number of cilia (Supplementary Fig. S1).

## Discussion

The molecular diagnosis of ciliopathies, such as BBS, can be difficult due to the genetic heterogeneity associated with these disorders. The combination of several genetic approaches allowed us to confirm the molecular diagnosis of five BBS patients whereby seven predicted pathogenic variants, some of them novel, were found in three *BBS* genes: *BBS1*, *BBS5* and *BBS6*. While *BBS1* is the most frequently mutated gene in BBS, accounting for 23% of cases and 15% of the total mutational load^10,26^, *BBS5* represents a minor contributor, with only 2% of cases.

The most common disease-causing mutation in *BBS1*, p.Met390Arg, was first identified by the genotyping microarray in heterozygous state in patient GBB27, for whom a second heterozygous change in this gene was found by direct sequencing, p.(His504Hisfs*48), a small deletion of 11 nucleotides assumed to be pathogenic. This genotype is consistent with other cases in which the presence of heterozygous p.Met390Arg variant was generally associated with another mutated allele in the same gene^27^. The other two *BBS1* homozygous changes were: p.(Trp23*), a nonsense mutation located in the first residue of the N-terminal domain of the encoded protein, and p.(Val366Asp), which lies in a WD40 repeat domain, usually required for the assembly of multiprotein complexes^28^. Regarding the three *BBS5* variants, one of them was novel, p.(Phe180Phefs*8), and found in the same patient (RTP23) in compound heterozygosity with the missense change p.(Arg138Cys), which was classified as novel at the moment of identification since it was not found in public databases. A third *BBS5* mutated allele, p.(Asn184Ser), was found in heterozygous state in a patient (RP1573) who in turn resulted homozygous for the novel *BBS6*:p.(Gly411Ala) variant. Other families with heterozygosity for p.(Asn184Ser) have been reported^29,30^, one of them also homozygous for *BBS1*:p.Met390Arg mutation^29^. The Asn184 residue, like Phe180, localizes in a putative domain called DM16, a predicted repeat domain evolutionary conserved across species. Therefore, mutations in this domain are thought to be pathogenic^30^. Many researchers have proposed a complex mode of inheritance in BBS involving the presence of three mutated alleles in two different *BBS* genes, in which the third allele would also be necessary for the development of the disease (triallelism), or would exert a modifying or epistatic but not causal effect, leading to more severe phenotypes^29,31^. In this case, there were no siblings available to evaluate the specific effect of this allele, but according to reported cases, it seems to be a genetic modifier.

Although several evidences suggest that the identified variants are likely to be pathogenic, and considering that BBS proteins, as many others related to cilia, are highly conserved across ciliated organisms and share a high protein identity with zebrafish orthologs^32^, we performed functional analysis in zebrafish model to assess their effects and validate their pathogenicity. Zebrafish model has been widely used to analyse ciliary dysfunction^32^, with several reported models of BBS, but also of other ciliopathies like Meckel-Gruber syndrome^33,34^, Joubert syndrome and nephronophthisis, predominantly showing defective melanosome transport, retinal defects, defective cilia in KVs and kidney anomalies, among others^25,32,35^.

Thus, the injection of MOs against *bbs1*, *bbs5* and *bbs6* genes in zebrafish embryos confirmed the previously reported phenotypes, including several developmental defects such as curved and/or shortened body axis, together with notochord and somite anomalies^36^, which are also commonly associated with other ciliopathies. Thus, *Bbs* disruption causes phenotypes associated with planar cell polarity (PCP) defect. Using the same MOs, Zaghloul *et al*. (2010) also reported that the BBS disruption causes phenotypes associated with planar cell polarity (PCP) defects in zebrafish; likewise, there are other studies that also showed evidence for similar axial phenotypes by *in vivo* loss of function methods in zebrafish^21,22^. However, other recent studies^37^ do not report early developmental phenotypes typically associated with PCP pathway defects. These possible discrepancies could be attributed to behavior/mutagenic efficiency of the different MOs used. Our *bbs* phenotypes were efficiently rescued by the corresponding WT human *BBS* capped-mRNAs, which confirms the specificity of the observed defects, however new mutant BBS variants mRNAs led to a high number of embryos with an abnormal phenotype, providing clear evidences of their pathogenicity. In addition, morphant embryos also displayed KV abnormalities, mainly concerning cilia length and number. KV has been proposed as a transient ciliated organ of asymmetry with a key role in left-right (LR) patterning by the establishment of a directional fluid flow^26,38–40^, a function equivalent to the mouse node^40,41^. KV originates during gastrulation and acquires a spherical pattern visible in the tail-bud during the somite stages^32^. It is known that the disruption of KV represents the first morphological abnormal phenotype associated with *bbs* gene KD^2,26,32^, that is why KV has been very useful in functional assessment of ciliopathies.

We focused on KV cilia length since it has been demonstrated that it is less variable than KV cilia number and lumen size^40^, and an optimal cilia length is required for proper KV functional flows^39,40^. We found that all genetic changes, with the exception of p.(Phe180Phefs*6), failed to rescue the cilia shortening caused by their respective gene conditional KD, therefore confirming that the tested *bbs* gene variants are deleterious for protein function, as reported by other authors for *bbs* gene KD^32,36,42,43^. In particular, the only nonsense change included in this study, *BBS1*:p.(Trp23*), resulted in the most reduced cilia length, less than half of the cilia length of control embryos and even shorter than in bbs1-MO injected embryos, which was also consistent with the severity of the phenotype observed by WISH. Despite the *BBS6* missense variant (p.Gly411Ala)) resulted in a smaller reduction of cilia length compared to controls, it was also statistically significant, relatively similar to bbs6-MO length and consistent with the corresponding morphant phenotype, classifying it as null. This variant also showed a significant reduction in the number of KV cilia. The p.(Asn184Ser) allele was already analysed by *in situ* in zebrafish embryos and classified as null in a previous study^24^, but it was included here to evaluate its effect on KV cilia length, resulting in a significant shortening. This, together with the observed gastrulation defects, also made us classify it as null. Thus, the results of *BBS5*:p.(Asn184Ser) and *BBS6*:p.(Gly411Ala) changes, found in the same patient (RP1573) in heterozygous and homozygous state, respectively, could justify the development of a more severe clinical spectrum of all the patients included in this study, which represents a clear example of complex inheritance.

In view of these results, *bbs1* morphants seem to display the most severe early developmental phenotypes with clear somitic and notochordal defects, which are also reflected in the most significant KV cilia shortening. Given that mutations in *BBS1* are one of the most common causes of BBS syndrome, some authors had already suggested that BBS1 should play an important role^43^. Thus, it has been demonstrated that BBS1 is a key regulator of BBSome entry into cilia^8^.

In general, the three mutated genes analysed in our study, as other *BBS* genes, have been well characterized not only in zebrafish, but also in mice and *in vitro* ciliated cells. Bbs1- and Bbs6-knockout in mice led to phenotypes similar to those observed in Wnt zebrafish mutants^44^. The importance of the chaperonin complex in the assembly of the BBSome has been demonstrated in mice, pointing out that Bbs6 is necessary for the stability of Bbs2 and Bbs7^45^. In fact, a missense variant in this gene resulted in a markedly decreased interaction of BBS6 with BBS2, one of the BBSome components, which in turn represents the second subunit being incorporated into the complex^15^. Mutations in *BBS6* are also associated with McKusick-Kaufman syndrome^46^, another ciliopathy characterized by congenital heart defects, hydrometrocolpos and postaxial polydactyly, one of the primary features of BBS. Moreover, many researchers found that the suppression of *BBS1* and *BBS5* dramatically affects to the ability of retinal pigment epithelium (RPE) cells to ciliate^13,14^, consistent with the studies showing KV abnormalities in *bbs1*, *bbs5 and bbs6* KD embryos, suggesting a key role in the formation of KV^32,42^.

Over the last decade, many efforts have been made to gain insight into the ciliary functions. Thus, it is known that cilia are involved in several developmental pathways, such as Shh, key for tissue morphogenesis, and Wnt signalling, whose proper functioning is necessary for organ growth and differentiation^47^. In this sense, zebrafish has been a useful tool in the study of the connection between ciliary dysfunction and ciliopathies. The possibility to carry out genetic interaction analyses in zebrafish has revealed the role of the BBSome in the PCP, also named non-canonical Wnt, pathway^21^. *BBS* genes also seem to be involved in the regulation of the canonical Wnt pathway^22^. Additionally, zebrafish model has also revealed the involvement of *BBS* genes in Shh pathway and, therefore, its usefulness in assaying human polydactyly, one of the most frequent clinical features in BBS patients and typically associated with Shh pathway defects^17,42^, which could not be directly assayed in mice^48^. All this reflects the great importance of *BBS* genes in development and the need of an unaffected BBS function for an appropriate maintenance of cilia and ciliated cells^32^.

In summary, our results highlight the efficiency of the genetic strategy followed here for the molecular diagnosis of five families with clinical spectrum of BBS, allowing us to identify candidate pathogenic changes in known *BBS* genes. Functional assays in zebrafish provide clear evidences of the pathogenic effect of the analysed variants. Although certain *in silico* deletions and nonsense changes are usually considered as pathogenic without performing further functional analysis, this *in vivo* study confirms their harmful effects. Likewise, in the case of the missense variants the present experiments provided evidence for their implication in the phenotype. Despite the major advances in genomic engineering have provided new approaches to generate genetic mutants for experimental studies, such as zinc-finger nucleases (ZFNs), transcription activator-like effector nucleases (TALENs), and CRISPR/Cas-based genome editing systems, we show that the use of MOs to transiently KD *BBS* genes in zebrafish is a useful, efficient and relatively rapid approach to assay variant pathogenicity and model human diseases like BBS and other ciliopathies.

## Methods

### Study cohort

Five patients from unrelated families clinically diagnosed with BBS were included in this study. All of them are of Spanish origin, except one patient from India. A fifth degree of consanguinity was referred for one of the Spanish families (RP1573). This study was approved by the Galician Ethical Committee for Clinical Research (Spain - no.2006/08) and adhered to the tenets of the Declaration of Helsinki. Informed consent was obtained from all patients or their guardians.

### Mutational analysis

Peripheral blood from all participants and available family members was collected for DNA extraction using the Flexigene DNA kit 250 (Qiagen), following the manufacturer’s protocol. Mutations in three of these families were described in previous work^23^, while those in the remaining two families were identified by homozygosity mapping (RP1573) and WES (RTP23) (unpublished results). The functional consequences of missense changes were predicted using PolyPhen2^49^, SIFT^50^, Pmut^51^ and Mutation Taster^52^ tools. Nomenclature of novel variants was done according to the guidelines of Human Genome Variation Society (http://www.hgvs.org/).

Sanger sequencing was performed in probands to validate the selected changes from WES data and to analyse coding regions of the candidate gene identified by homozygosity mapping. Then, segregation analysis was carried out in available family members. We also analysed 100 control chromosomes of unrelated Galician individuals, to assess the allele frequency of novel variants, which were also were checked against the following databases: Exome Aggregation Consortium (ExAC, http://exac.broadinstitute.org/), dbSNP (https://www.ncbi.nlm.nih.gov/projects/SNP/) and HGMD (Human Gene Mutation Database, http://www.hgmd.cf.ac.uk/ac/index.php).

### Site-directed mutagenesis

cDNAs encoding full length human *BBS* genes cloned into pCS2 + vector, which were provided by Dr. Norann Zaghloul (University of Maryland School of Medicine), were used to synthesize capped mRNAs for zebrafish rescue experiments. Site-directed mutagenesis was performed using the QuickChange® II XL Site-Directed Mutagenesis kit (Agilent Technologies) to introduce sequence variants of unknown functional significance into the corresponding WT cDNAs, and confirmed by direct sequencing. The primers used are shown in Supplementary Table S1.

### *In vitro* transcription-translation assay

Expression and translation stability of mutated human BBCs proteins (from RNA transcripts) from their DNA constructs was assessed in an *in vitro* transcription and translation-coupled system (TNT^®^ Coupled Reticulocyte Lysate Systems; Promega). The amplified full length mutated and non-mutated BBS cDNAs were subcloned into the pCS2 + vector. The pCS2 + vector utilizes the SP6 RNA polymerase binding site that can be used directly for *in vitro* transcription–translation assays. The assay was performed according to the instructions provided with the Promega TNT Coupled Reticulocyte Lysate Systems using Transcend^TM^ non-radioactive translation detection system, (Promega). The synthesized proteins were analyzed by SDS gel electrophoresis on a 12% acrylamide gel. To determine whether all mutant proteins are specifically recognized, western blot analysis and detection of the corresponding biotinylated proteins from the TNT^®^ assay was performed using Streptavidin-HRP Conjugate and Transcend^TM^ Chemiluminescent Substrate as described in the manufacturer protocol.

### Zebrafish husbandry

Fish (Zebrafish, *Danio rerio*) embryos were obtained as previously described^53^ and staged according to Kimmel *et al*.^54^. Experiments were performed with a TU-WT strain (Tuebingen (TU), Nüsslein-Volhard Lab). Dechorionated embryos were collected for *in vivo* imaging or fixed overnight at 4 °C in 4% paraformaldehyde (PFA) in 1X PBS, washed in 1X PBS, and dehydrated through methanol series and stored at −20 °C in 100% methanol for *in situ* hybridization. Ethical approval (Ref: CSIC/OH-150/2014) was obtained from the Institutional Animal Care and Use Committee of the IIM-CSIC Institute in accordance with the National Advisory Committee for Laboratory Animal Research Guidelines licensed by the Spanish Authority (RD53/2013) and conformed to European animal directive (2010/63/UE) for the protection of experimental animals.

### Morpholino knockdown and mRNA rescue

Translational start blocking MOs (GeneTools, LLC) were used to specifically knockdown *bbs1*, *bbs5* and *bbs6* endogenous zebrafish genes (Supplementary Table S2)^24^. The MOs were resuspended in water to a final concentration of 20 mM (stock solution). For rescue assays, capped mRNAs were transcribed *in vitro* using the mMessage mMachine SP6 transcription kit (Ambion) and each human *BBS1*, *BB5 or BBS6* cDNA (WT or mutated) cloned into pCS2 + vector were used as templates. For zebrafish embryo injections, 2 nl of each specific *bbs*-MO and capped human mRNA solution (Supplementary Table S2) were injected into one- or two-cell stage embryos with 1% of phenol red as a tracer, based on amounts previously reported in the literature^24^. About 180–300 embryos per condition were injected. Microinjection was performed under a dissection microscope (MZ8, Leica) fitted with a MPPI-2 pressure injector (ASI Systems).

### Whole-mount *in situ* hybridization (WISH)

Whole-mount *in situ* hybridization was carried out on zebrafish embryos previously fixed at 8–12 somite stage (ss) in 4% paraformaldehyde in 1X PBS, using a cocktail of three digoxigenin-labeled antisense RNA probes for *pax2*, *myoD* and *krox20*, according to previously described protocols^55–57^. Images of flat-mounted and cleared embryos were taken using a Leica M165FC stereomicroscope with DFC 310 camera at 10X magnification, in order to analyse the morphological defects in early-development of injected embryos.

### Whole-mount Immunofluorescence and cilia analysis

Zebrafish control and injected embryos were fixed at 8–12 ss, for KV analysis, in 4% PFA (Sigma) for 24 h at 4 °C, then washed in 1X PBS- 1% Triton X-100 (PBST; Sigma) and dehydrated gradually through a methanol series and stored in 100% methanol (Sigma) at −20 °C. Whole-mount immunofluorescence experiments were performed as follows: embryos were first washed with PBST several times, blocked with goat serum 5% for 1 h before incubation with primary and secondary antibodies both in PBS-Triton (1%) plus 2% goat serum overnight at 4 °C. We stained KV cilia using a mouse anti-acetylated α-tubulin (1:750; T7451, Sigma) monoclonal antibody and, subsequently, with Alexa Fluor 594-conjugated goat anti-mouse secondary antibody (1:1000, ThermoFisher). Nuclei were stained with DAPI.

Finally, embryos were mounted in 75% glycerol and confocal images were acquired on a Leica SP5 microscope and analysed using ImageJ software. Identical settings (gain, offset, laser power) were applied to all z-stack acquisitions, and all fluorescence measurements were performed on maximum intensity projection, also calculated with ImageJ.

### Statistical analyses

Statistics for KV cilia length measures were calculated using one-way ANOVA paired with the Welch significant difference test in SPSS v22.0 statistics software (IBM). To compare cilia number, we performed a Mann-Whitney test for non-parametric data. A p-value of <0.05 was consider as statistically significant.

## Supplementary information

Supplementary Information
