# Author Correction: Functional analysis of new human Bardet-Biedl syndrome loci specific variants in the zebrafish model

**DOI:** 10.1038/s41598-020-60006-5

**Published:** 2020-02-13

**Authors:** Sheila Castro-Sánchez, Paula Suarez-Bregua, Rossina Novas, María Álvarez-Satta, Jose L. Badano, Josep Rotllant, Diana Valverde

**Affiliations:** 1 Grupo de Biomarcadores Moleculares, Departamento de Bioquímica, Genética e Inmunología, Facultad de Biología, Universidad de Vigo, Lagoas-Marcosende s/n, 36310 Vigo, Spain; 2 Grupo de Investigación en Enfermedades Raras y Medicina Pediátrica, Instituto de Investigación Sanitaria Galicia Sur (IISGS), Vigo, Spain; 3 Centro de Investigaciones Biomédicas (CINBIO), Centro Singular de Investigación de Galicia 2016–2019, Universidad de Vigo, Vigo, Spain; 4 Department of Biotechnology and Aquaculture, Institute of Marine Research, Spanish National Research Council (IIM-CSIC), Vigo, Spain; 5 Human Molecular Genetics Laboratory, Institut Pasteur de Montevideo, Mataojo 2020, Montevideo, CP11400 Uruguay

Correction to: *Scientific Reports* 10.1038/s41598-019-49217-7, published online 10 September 2019

In this Article, images E and F in Figure 5 are incorrect. The correct Figure 5 appears below as Figure 1.

**Figure 1:**
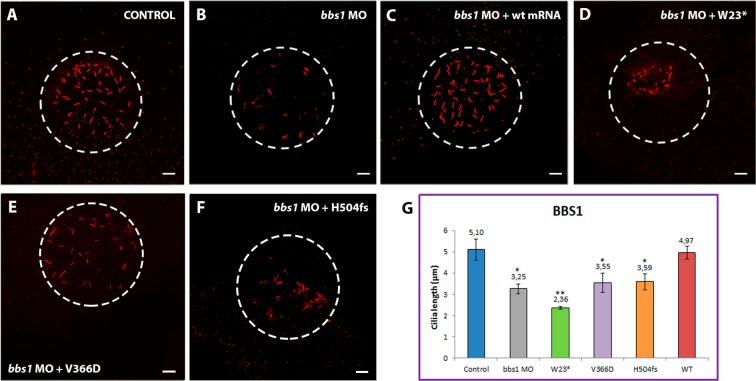
.

