# Supplementary material for: Functional analysis of new human Bardet-Biedl syndrome loci specific variants in the zebrafish model
Source: Sci Rep. 2019 Sep 10;9:12936. doi: 10.1038/s41598-019-49217-7 (PMC6736949; doi:10.1038/s41598-019-49217-7)
Supplement: Supplementary file 1 — Supplementary Information [file 41598_2019_49217_MOESM1_ESM.pdf]

Functional analysis of new human Bardet-Biedl syndrome loci specific variants in the zebrafish model

Sheila Castro-Sánchez[1,2,3], Paula Suarez-Bregua[4], Rossina Novas[5], María Álvarez-Satta[1,2,3], Jose L. Badano[5], Josep Rotllant[4], Diana Valverde[1,2,3]*

[1] *Grupo de Biomarcadores Moleculares, Departamento de Bioquímica, Genética e Inmunología, Facultad de Biología*, *Universidad de Vigo*, Lagoas-Marcosende s/n, 36310 Vigo, Spain.

[2] *Grupo de Investigación en Enfermedades Raras y Medicina Pediátrica*, *Instituto de Investigación Sanitaria Galicia Sur* (IISGS), Vigo, Spain.

[3] *Centro de Investigaciones Biomédicas (CINBIO), Centro Singular de Investigación de Galicia 2016-2019*, *Universidad de Vigo*, Vigo, Spain.

[4] Dept of Biotechnology and Aquaculture, Institute of Marine Research, Spanish National Research Council (IIM-CSIC), Vigo, Spain.

[5] Human Molecular Genetics Laboratory, Institut Pasteur de Montevideo, Mataojo 2020, Montevideo, CP11400, Uruguay.

*Corresponding authors:

Diana Valverde, PhD & Sheila Castro, PhD

Departamento de Bioquímica, Genética e Inmunología, Facultad de Biología, Universidad de Vigo, Lagoas-Marcosende s/n, 36310, Vigo, Spain.

dianaval@uvigo.es / shey.cs9@gmail.com

Phone: 0034 986 811 953

Table S1. Primer sequences for site-directed mutagenesis.

GENE	VARIANT	EXON	SEQUENCE (5' →3')	ANNEALING TEMPERATURE (ºC)
BBS1	c.68G>A/<br>p.(W23*)	2	F: GAGGCCAATTCGAAGTAGTTGGATGCGCACTAC<br>R: GTAGTGCGCATCCAACTACTTCGAATTGGCCTC	108.5
BBS1	c.1097T>A/<br>p.(V366D)	11	F: CAAGGCCCTGCTCAATGACATCCACACCCCGGATG<br>R: CATCCGGGGTGTGGATGTCATTGAGCAGGGCCTTG	83.2
BBS1	c.1510_1520delCACCTGCAGAA/<br>p.(H504H*fs**48)	15	F: CCCCACCTTTAAGCTCACACTT-----------CACCTCAACAAC<br>R: GTTGTTGAGGTG-----------AAGTGTGAGCTTAAAGGTGGGG	82.2
BBS5	c.412C>T/<br>p.(R138C)	6	F: CACAGAGCTTATGAAACTTCTAAAATGTATTGTGATTTTAAATTAAGAAGTGCAC<br>R: GTGCACTTCTTAATTTAAAATCACAATACATTTTAGAAGTTTCATAAGCTCTGTG	79.3
BBS5	c.538delT/<br>p.(F180F*fs**6)	7	F: GTGATCAGGGCAATTTAGGAACC-TTTTTATTACCAATGTGAGAATT<br>R: AATTCTCACATTGGTAATAAAAA-GGTTCCTAAATTGCCCTGATCAC	81.2
BBS5	c.551A>G/<br>p.(N184S)	7	F: GGCAATTTAGGAACCTTTTTTATTACCAGTGTGAGAATTGTGTGGCAT<br>R: ATGCCACACAATTCTCACACTGGTAATAAAAAAGGTTCCTAAATTGCC	73.9
BBS6	c.1232G>C/<br>p.(G411A)	5	F: GGCTTTGTTGGGAGGTGCCTGTACTGAAACTCATT<br>R: AATGAGTTTCAGTACAGGCACCTCCCAACAAAGCC	79.3

These primers were designed by using the Quick Change Primer Design software (Agilent Technologies; http://www.genomics.agilent.com/primerDesignProgram.jsp?_requestid=1174494). The change introduced in each sequence is highlighted in red. F: forward primer; R: reverse primer.

Table S2. Morpholino sequences for each *bbs* gene and injected doses of morpholinos and capped mRNAs.

GENE	MO sequence	MO concentration (ng/nl)	mRNA concentration (pg/nl)
bbs1	5’-GGCTGGCAAATAAGCTGTCCACAG-3'	8.0	100
bbs5	5’-GTCCAACACCGACGCCATGATCACT-3'	4.0	50
bbs6	5’-GCTTCTTCTTACTAATGCGAGACAT-3'	3.0	100

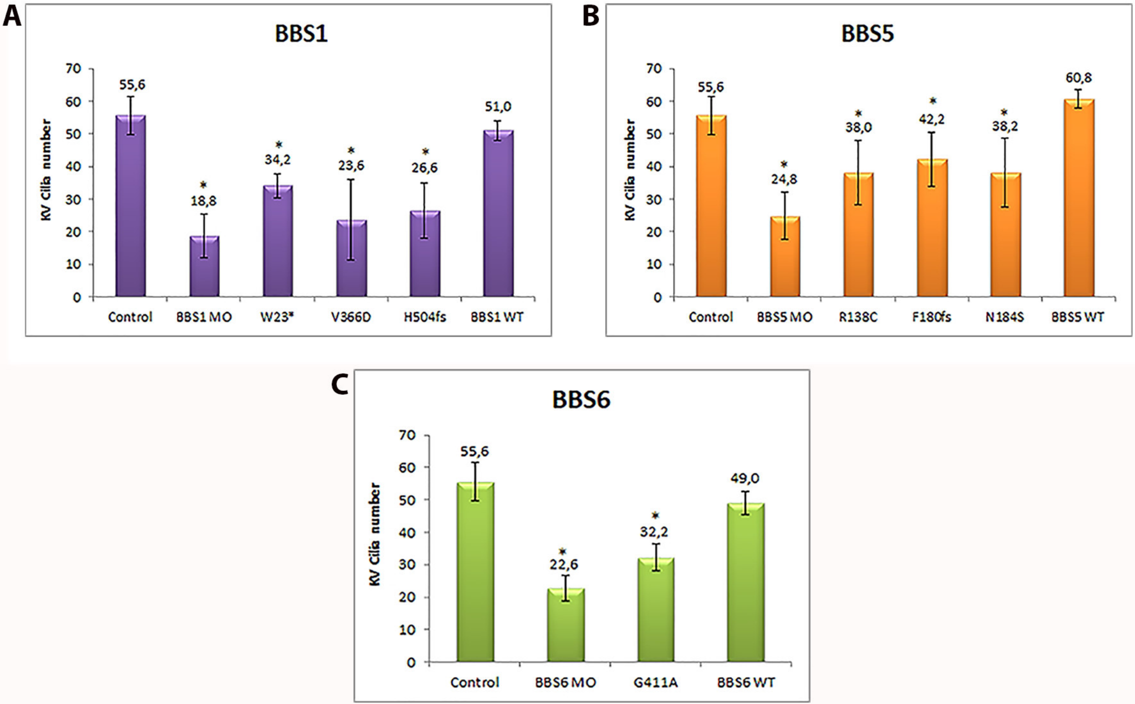

Supplementary Fig. S1. Quantification of KV cilia number in BBS1 (A), BBS5 (B) and BBS6 (C) conditions compared to controls (non-injected embryos). (*) $p<0.01$

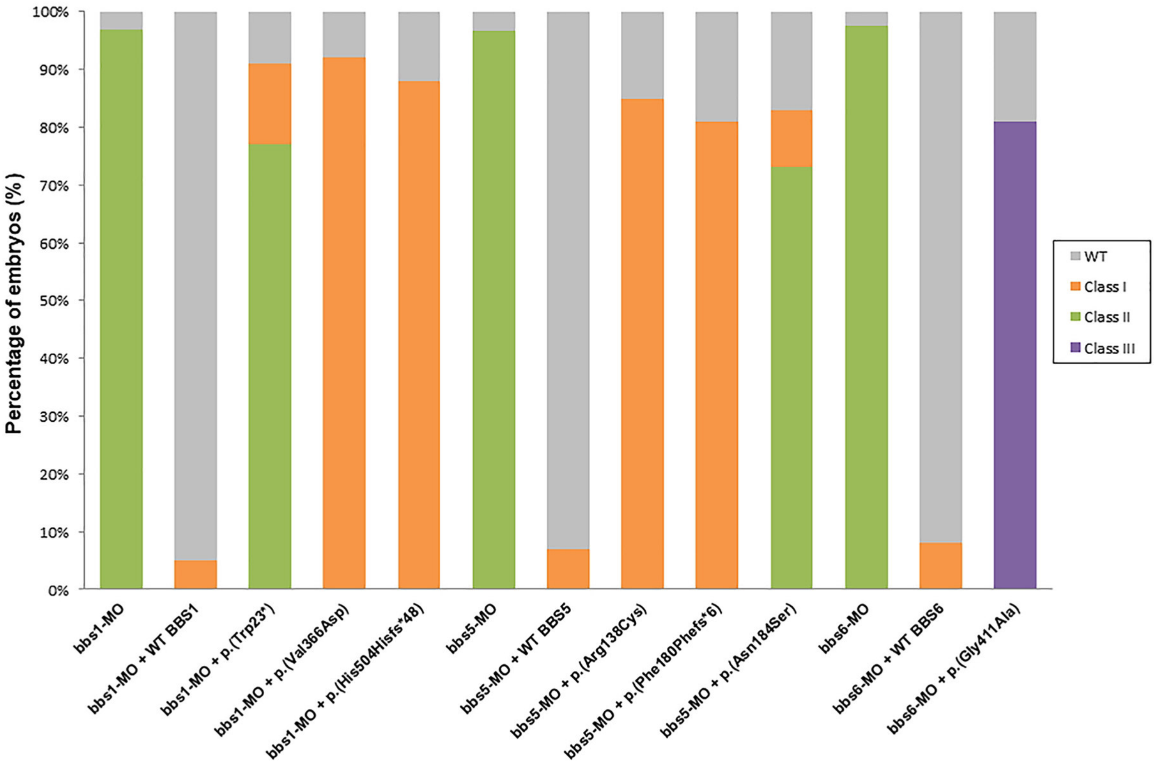

Supplementary Fig. S2. Quantification of degree of bbs-MO rescue by human BBS mRNAs analyzed by in situ hybridization of myoD/krox20/pax2 gene markers in zebrafish embryos (n=60-75). None of the human mutant BBS variants were able to entirely recover the zebrafish knockdown bbs phenotypes (Class I: morphant phenotype partially recovered but more severe than the WT phenotype; Class II: morphant-like phenotype; Class III: more severe phenotype than the morphant one).
